# National and subnational coverage and inequalities in reproductive, maternal, newborn, child, and sanitary health interventions in Ecuador: a comparative study between 1994 and 2012

**DOI:** 10.1186/s12939-020-01359-1

**Published:** 2021-01-28

**Authors:** Paulina Rios Quituizaca, Giovanna Gatica-Domínguez, Devaki Nambiar, Jair Licio Ferreira Santos, Stefan Brück, Luis Vidaletti Ruas, Aluisio J.D. Barros

**Affiliations:** 1 Central University of Ecuador, Faculty of Medicine, Quito, Ecuador; 2 Riberao Preto Medical School, University of Sao Paulo. FMRP-USP, São Paulo, Brazil; 3 International Center for Equity in Health, Universidade Federal de Pelotas, Pelotas, Brazil; 4 The George Institute for Global Health, Delhi, India; 5 Central University of Ecuador, Faculty of Biological Sciences, Quito, Ecuador

**Keywords:** Healthcare disparities, Maternal- child health services, Reproductive health services, Continuity of patient care, Socioeconomic factors, Health care surveys, Cross- sectional studies

## Abstract

**Background:**

Latin America (LA) has experienced constant economic and political crises that coincide with periods of greater inequality. Between 1996 and 2007 Ecuador went through one of the greatest political and socio-economic crises in Latin America, a product of neo-liberal economic growth strategies. Between 2007 and 2012 it regained political stability, promoted redistributive policies, and initiated greater social spending. To understand the possible influence on the political and economic context, we analyzed the coverage and inequalities in five Reproductive, Maternal, and Child Health (RMNCH) and two water and sanitation interventions using survey data from a broad time window (1994–2012), at a national and subnational level.

**Methods:**

The series cross-sectional study used data from four representative national health surveys (1994, 1999, 2004 and 2012). Coverage of RMNCH and sanitary interventions were stratified by wealth quintiles (as a measure of the socio-economic level), urban-rural residence and the coverage for each province was mapped. Mean difference, Theil index and Variance-weighted least squares regression were calculated to indicate subnational and temporal changes.

**Results:**

From 1994 to 2004, Ecuador evidenced large inequalities whose reduction becomes more evident in 2012. Coverage in RMNCH health service-related interventions showed a rather unequal distribution among the socioeconomic status and across provinces in 1994 and 2004, compared to 2012. Sanitary interventions on the contrary, showed the most unequal interventions, and failed to improve or even worsened in several provinces. While there is a temporary improvement also at the subnational level, in 2012 several provinces maintain low levels of coverage.

**Conclusions:**

The remarkable reduction of inequalities in coverage of RMNCH interventions in 2012 clearly coincides with periods of regained political stability, promoted redistributive policies, and greater social spending, different from the former neo-liberal reforms which is consistent with observations made in other Latin American countries. Territorial heterogeneity and great inequalities specially related with sanitation interventions persists. It is necessary to obtain high quality information with sharper geographic desegregation that allows to identify and understand local changes over time. This would help to prioritize intervention strategies, introduce multisectoral policies and investments that support local governments.

## Key messages:

- In Ecuador, the reduction of inequality in health service- related RMNCH interventions coincides with periods of political stability, promoted redistributive policies, and greater social spending, but inequalities in sanitation interventions remain high.
- While there is a temporary improvement also at the subnational level, in 2012 several provinces maintain low levels of coverage, related to local determinants that must be identified and analyzed to improve interventions in a fairer way.

## Background

Inequality is a major global challenge, including in Latin America, which has experienced periods of dramatic decline in levels of social spending accompanied by economic crises and political instability [1]. The widespread adoption of neo-liberal economic growth strategies has increased poverty and widened income inequalities in the provision of health services [2, 3]. In countries such as Ecuador, Bolivia, Argentina and Brazil, periods of greatest economic and political crisis coincide with marked increases in inequality, as measured by the Gini Index [4, 5].

Initiatives like Countdown to 2015 and 2030 have placed emphasis on Reproductive, Maternal, Neonatal and Child Health (RMNCH) interventions globally, relying on publicly available Demographic and Health Surveys. Over the past two decades, several Latin American countries have reported increasing coverage of essential RMNCH interventions [6, 7]. To what extent larger economic and political crises have impacted coverage has been scarcely studied [8–10]. Moreover, the distribution of coverage across population subgroups remains understudied; and more needs to be done to identify underserved groups, and (re) allocate resources according to varying needs [8, 11]. Further, based on how interventions are faring across population groups, they may need to be integrated, reducing costs, duplication, and inefficiencies [12].

Ecuador is among those Latin American countries that endured a long period of political and socio-economic crisis during the 1990s and 2000s. From 1996 to 2006, the country had a troubled economy and unstable governance. This peaked with a hyperinflation in 2000, followed by ‘dollarization’ and liberalization of fiscal policies [13–15]. Between 1998 and 2004, Ecuador’s modified Gini index increased from 49.7 to 53.9, the highest value ever seen in the country’s history; accompanied by a consistent increase in out-of-pocket expenditure through the year 2006 [13, 16, 17]. In 2004, access to health services was found to be constrained among people with low-income households, indigenous population and inhabitants of rural areas in comparison to higher income, non-indigenous and urban populations [18, 19].

During the subsequent period - 2006 to 2012- however, there were drastic changes in the country: the prevalence of poverty declined from 37.6 to 27.3%; the gross domestic product (GDP) increased from 4.2 to 12.6% [16], and the Gini index fell from 52.2 to 46.1. Total health expenditure as a percentage of GDP increased from 5.9 in 2006 to 9.2 in 2014 [17]; out-of-pocket expenses reduced significantly [16] given a notable increase in public investment in health. As a result of these features, Ecuador rose from the 111th rank in 2000 to the 13th in 2014 according to the World Health Organization’s (WHO) Health Care Efficiency (HCE) Index [20, 21].. Between 2006 and 2014 inequalities in access to health services by socio-economic status decreased [22].

Existing studies have compared health inequalities in shorter periods of time [22–24], and there remains a lack of scientific evidence about inequalities in intervention coverage that reflects the changing macroeconomic and political contexts, particularly through analyses that examine subnational level. Using data from four national health surveys in Ecuador, we describe inequalities in coverage of RMNCH and sanitary interventions during and after periods of marked political, economic, and social crisis, between 1994 to 2012.

## Materials and methods

### Data sources and selection of indicators

The present study is a series cross-sectional analysis using nationally representative surveys. For the years 1994, 1999 and 2004, we analyzed data from the Reproductive Health Surveys (RHS), which provide data on women of childbearing age from 15 to 49 years (13,582; 14,285 and 10,814, women for each survey respectively), children under 3 and 5 years (8837; 8691 and 4184 children for each survey respectively) and information on household assets (14,084; 19,896 and 10,985 households for each survey respectively) [25–27]. The 2012 survey included 18,213 women from 15 to 49 years; 19,949 homes and 10,098 children under 5 years of age [28]. More information is available in official reports which are publicly available [29]. RHS was not available after 2004, so we relied on the national survey of health and nutrition (ENSANUT) of 2012 [28], which had comparable indicators.

We selected intervention indicators that are considered essential across the continuum of RMNCH care at the community level [9] and used in global Sustainable Development Goal (SDG) monitoring efforts, such as the Countdown to 2030 initiative [30, 31]. We further identified interventions related to access to safe water and improved sanitation (WAS), which are established in the existing literature as being highly complementary to RMNCH, and are associated with great gains in mother and child survival [32]*.*

All indicators were operationalized based on standardized definitions of coverage of interventions [33] which represent the proportion of individuals who access the intervention at the national level and by subgroups, with their respective standard error; these indicators were constructed in simple Microsoft Excel sheets from both surveys (RHS and ENSANUT) ensuring comparability across datasets and time periods. The definitions of the indicators, their numerator and denominator are presented in the Supplementary annex 1.

Comparability of indicators over time is a well-known challenge in studies that use household surveys, due to differences in data collection and instrumentation [34, 35]. This study analyzes seven temporally comparable indicators in a representative sample while considering the disaggregation by population subgroups. Five were **RMNCH indicators**: *Use of modern contraceptive, Antenatal care (4+ visits), Institutional delivery, Early initiation of breastfeeding, Full immunization;* and two were Water and Sanitation (**WAS) indicators:** *Improved sanitary facility, Improved drinking water source*.

### Dimensions of inequality

Socioeconomic status was assessed in this study using an asset index, following the convention used in various Low and Middle-Income Countries (LMICs) [7, 35–37]. Indicators on active assets of the selected households, materials used for housing construction and types of water and sanitation access facilities were analyzed using Principal Component Analysis (PCA) to generate the wealth score for each household. Each individual was classified according to the total score of the family in which s/he resided and finally, the sample was classified and divided into quintiles with the Quintile 1 (Q1) representing the 20 poorest percent and Quintile 5 (Q5) representing the 20 wealthiest percent of households [36, 38]. The same methodology was applied for all four surveys. There were no marked demographic changes in the study period, which facilitated the interpretation of temporal trends [39].

We also disaggregated by place of residence (rural versus urban) and geographic region, as has been done for inequality analysis in several countries [40]. Following the definitions used in prior work [41], we compared intervention coverage among the population living in urban areas (those living in populated centers with 2000 or more inhabitants), to those living in rural areas (less than 2000 inhabitants). The subnational desegregation allows us to identify the sectors that achieved improvements and learn from the implemented actions and successful experiences that can be replicated, as well as identify neglected subpopulations prioritizing interventions in vulnerable sectors. The difference in the coverage between provinces for each year was considered in inequality analyses, as explained in the following section.

### Inequality analyses

#### Change in overall coverage over time on a national level

We calculated and plotted coverage and standard errors for each intervention. We used variance-weighted least squares regression to estimate the absolute annual change in intervention coverage [40].

For inequality analysis, we created *equiplot* graphs for the seven health interventions, showing the gap across wealth quintiles and between urban- rural residence over time, as done in earlier studies [7, 8, 10]. The equiplot is a data visualization tool that allows us to see all of the indicators and their level of coverage at the same time, providing a visual indication of absolute inequality over time [37, 42]. An analysis of complex inequality measures was carried out with data from the 2012 survey presented in the supplementary annex 2.

#### Change in coverage and inequalities over time by subnational level

We measured coverage of the five indicators across the four natural regions that make up Ecuador (Coast, mountain, Amazon and insular region). However, the Amazon region did not have a breakdown by province in 1994, so following the convention used in other studies [25–28], we calculated the coverage and their respective standard error for each province for only 3 years 1999, 2004 and 2012. Two summary measures were used: “mean difference from best” was an absolute measure for unordered inequality dimensions that calculates the mean difference in relation to the best coverage for each indicator and year (the higher the value, the greater the difference). The second, the Theil index, is a relative summary measure for unordered dimensions of inequality where “zero” may be interpreted as the absence of inequality and as the value becomes larger, inequality is greater [43].

We also measured the variation coverage over time and information for the provinces over the four-year survey periods was plotted to show the slope of change. Variance-weighted least squares regression was used to estimate the average of absolute annual change in the prevalence of each intervention, which allows us to consider the different time intervals between surveys (from 1994 to 2012), and to test the statistical significance of the observed trends [44]. The Moran Index was applied to the values of the regression coefficient, which helps to understand the degree to which one province spatially behaves similar to another (indicated by positive values), allowing the identification of autocorrelated patterns.

We used STATA (Stata corp.) version 15.0 for all analyzes, considering the design of the survey, such as sampling, grouping and stratification weights. The program R was used for plotting of the maps.

## Results

### Change in overall coverage over time

**Fig. 1:**
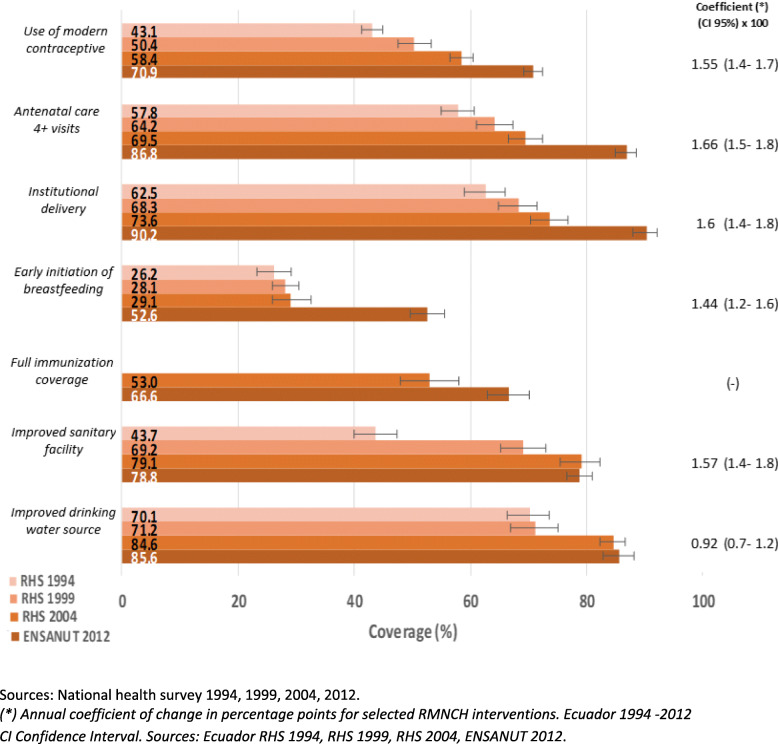
Coverage of RMNCH and WAS interventions. Sources: National health survey 1994, 1999, 2004, 2012. (*) Annual coefficient of change in percentage points for selected RMNCH interventions. Ecuador 1994–2012. *CI Confidence Interval. Sources: Ecuador RHS 1994, RHS 1999, RHS 2004, ENSANUT 2012*

Coverage of all the RMNCH interventions analyzed in the present study tended to increase significantly over the given period, and most drastically between 2004 and 2012 at the national level (see Fig. 1). The coverage of **health service- related RMNCH interventions** *(use of modern contraceptive, Institutional delivery, and antenatal care 4+ visits)* showed the highest slope values, with coverage progressively increasing at a rate of roughly 1.5 to 1.6% each year. The lowest coverage between 1994 and 2004 was of *early initiation of breastfeeding* with notable improvement between 2004 and 2012 (29.1 to 52.6%). In contrast, coverage of **interventions related to sanitation** showed a different pattern, where *improved sanitary facilities* showed an important progress from 43.7 to 69.2% between 1994 to 1999, but it did not show any progress between 2004 to 2012, while *Improved drinking water source* merely increased from 84.6% to 85.6 between 2004 to 2012.

### Socio-economic and geographic inequalities in coverage over time

**Fig. 2:**
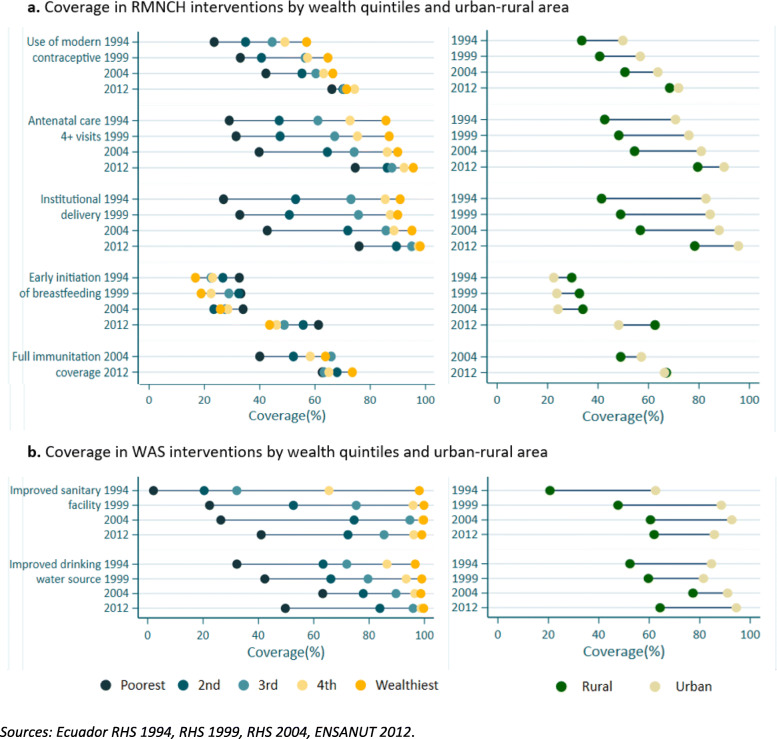
Coverage of intervention by Wealth quintiles and urban-rural area: 2**a**. RMNCH interventions 2**b**. WAS interventions. *Sources: Ecuador RHS 1994, RHS 1999, RHS 2004, ENSANUT 2012*

The gaps of the coverage of RMNCH interventions for each socioeconomic stratifier decreased over time (Fig. 2a). There was a remarkable difference in the reduction of inequalities between the period 2004 to 2012. Until 2004, a social gradient can be clearly observed, with a change in 2012 towards a pattern of marginal exclusion where all but the poorest quintile have reached reasonable levels of coverage. An inverse pattern of inequality was observed in the coverage of *early initiation of breastfeeding* such that the poorest quintile presented greater coverage*.*

The greatest inequality was observed in WAS interventions. Over time the population of the poorest quintile maintained low levels of about 50% coverage in basic services, indicating a wide gap compared to the richer population. Even in 2012, where *WAS* interventions show high level of national coverage, the disaggregation of information by wealth quintiles, demonstrates that still around half of the poorest quintile lacked coverage. (Fig. 2b). We quantified these inequalities in RMNCH and WAS interventions for 2012 data, where, rich people had 2.4 times greater coverage in *improved sanitary facilities* than the poorest (see Supplementary annex 2).

Figure 2 further indicates greater differences in coverage in favor of the population residing in urban areas, for all RMNCH interventions except for the breastfeeding intervention. Although coverage increased from 1994 to 2004 for all health service-related interventions the breach between rural and urban coverage remained about the same, most notably for institutional delivery which kept a difference of about 40%. These coverage differences decreased by at least half in 2012. These observed differences are even more accentuated considering WAS interventions. In Ecuador, there is a historical trend of poorer populations residing in rural areas [45], which we sought to understand more granularly using geographically disaggregated analyses.

### Subnational inequalities in coverage over time

**Fig. 3:**
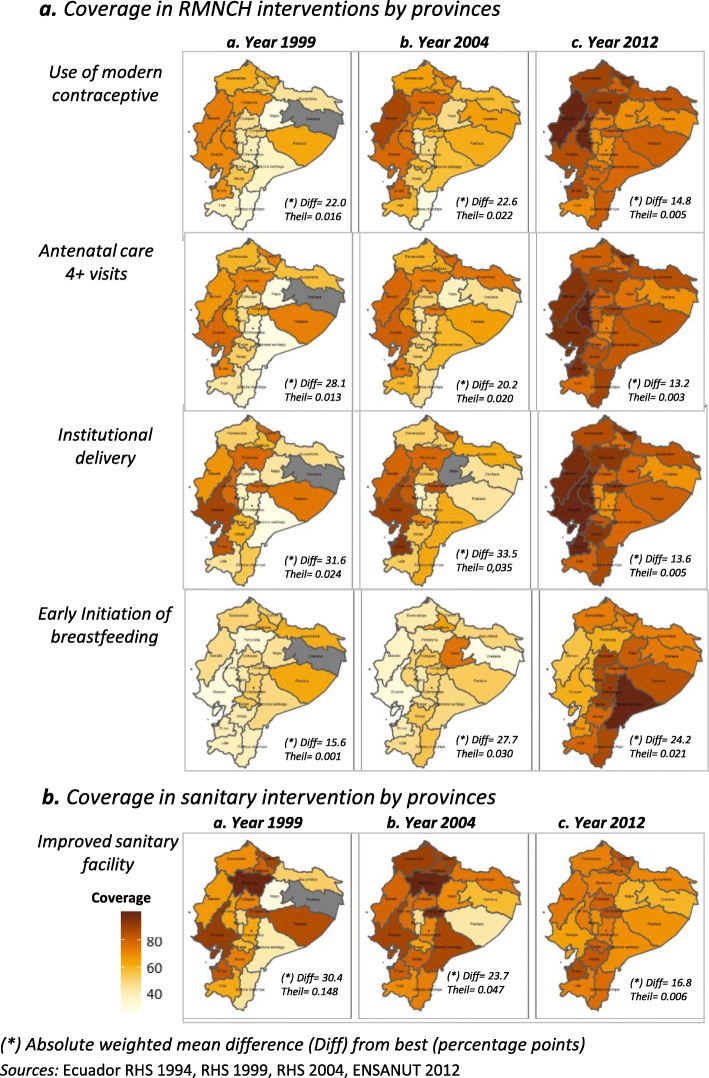
Differences between RMNCH and Sanitary interventions by provinces. Ecuador 1999–2012. (*) Absolute weighted mean difference (Diff) from best (percentage points). Sources: Ecuador RHS 1994, RHS 1999, RHS 2004, ENSANUT 2012

Disaggregated analysis by provinces allow us to explore whether changes in coverage were distributed equally between provinces at three points of time. High absolute weight mean differences (Diff) were observed in the years 1999 and 2004. *Institutional delivery* was the intervention with the greatest differences in the years 1999 and 2004 (Diff 1999 = 31.6, Diff 2004 = 33.5, Diff 2012 = 13.6) with evident improvements in coverage in 2012. Coverage of health services related interventions in 1999 and 2004 were more unequally distributed, with coverage becoming more equitable in 2012. The exception to this, as above, was *early initiation of breastfeeding* (Diff 1999 = 15.6, Diff 2004 = 27.7, Diff 2012 = 24.2), with poor coverage in the Ecuadorian coastal region. (see Fig. 3a). In sanitation coverage the weighted mean difference by province decreased over time (Diff 1999 = 30.4, Diff 2004 = 23.7, Diff 2012 = 16.8) (see Fig. 3b).

Average absolute annual change between all provinces – measured using variance weighted least squares regression - were positive and statistically significant in most of the measured interventions. Despite the notable improvements in the coverage of RMNCH interventions in several provinces of the Amazon region, coverage trailed other regions throughout all measured time periods. In Sanitation interventions in contrast, most of the coefficient values were low and not significant, meaning that the improvements between 1994 to 1999 were not sustained and even suffered a reduction of coverage in a few provinces for these indicators observing the period from 2004 to 2012. Neither RMNCH nor sanitary interventions presented hereby significant spatial autocorrelation applying Moran’s index. This means that no regional patterns were identified, with exception of early initiation of breastfeeding, with low coverage in almost all the coastal provinces of Ecuador (see Table 1 and details in supplementary annex 3 and 4).

**Table 1:** Average annual absolute change in percentage points by province and selected intervention. Ecuador 1994–2012

***Geographical Regions***	Province	A. Use of modern contraceptive	B. Antenatal care 4+ visits	C. Institutional delivery	D. Early initiation of breastfeeding	E. Improved sanitary facility
Cost	Guayas	1.3*	1.5*	0.9*	1.2*	0.5*
Cost	El Oro	1.3*	1.8*	1.2*	1.4*	1.4*
Cost	Manabi	1.8*	2.2*	2.4*	0	1.5*
Cost	Esmeraldas	2.0*	1.6*	2.3*	1.2*	1.2*
*Cost*	Los Rios	2.1*	2.7*	1.8*	1.1*	1.8*
Mountain	Pichincha	1.4*	1.1*	1.2*	1.5	0.1
Mountain	Imbabura	1.7*	1.2*	1.3*	1.5*	0.9*
Mountain	Carchi	1.9*	1.1*	1.0*	2.0*	0.4
Mountain	Tungurahua	1.9*	1.9*	1.4*	2.2*	0.9*
Mountain	Azuay	1.6*	2.1*	2.6*	1.9*	1.4*
Mountain	Canar	1.7*	1.6*	2.0*	2.1*	2.1*
Mountain	Bolivar	1.7*	2.2*	1.9*	2.4*	1.7*
Mountain	Chimborazo	1.6*	1.6*	3.1*	3.0*	1.4*
Mountain	Loja	1.7*	2.2*	2.3*	2.4*	1.9*
*Mountain*	Cotopaxi	1.8*	2.2*	2.8*	2.3*	1.8*
Amazon	Pastaza	1.2*	1.3*	1.7*	1.9	0.7
Amazon	Sucumbios	2.6*	1.6*	2.6*	1.1*	0.9
Amazon	**Morona santiago**	2.6*	3.6*	3.7*	3.9*	-1
Amazon	**Napo**	2.6*	3.4*	2.7*	2.4*	1.8*
Amazon	Orellana	0.5	2.6	5.5*	5.6*	0.2
*Amazon*	**Zamora Chinchipe**	3.5*	4.0*	2.6*	3.7*	1.7*
*Insular*	Galapagos	1.1*	1.4*	0.3	1	−0.2*
	**Moran Index**	- 0.047	−0.103	0.028	**0.143**^**ɫ**^	−0.036

(*) Statistically significant increase (positive values in percentage points) or decrease (negative values in percentage points) on coverage over time

*(*^**ɫ**^*) Coefficient statistically significant in the Moran Index*

## Discussion

This is the first study in Ecuador that explored the coverage and inequalities pertaining to seven essential RMNCH and sanitary interventions at national and sub-national levels in the period 1994–2012. During the decade from 1994 to 2004 Ecuador had large inequalities, with a general tendency towards a reduction in inequalities until 2012.

This is significant because Ecuador, like other countries in Latin America, has endured economic crisis and political instability through this period of time. Of all presidents democratically elected from 1970 to 2003 in the region, 23% were forced to leave their positions before the end of their terms and 40% faced challenges in their tenure [4, 46]. In 2004, the percentage of poor in Latin America reached 46.9% [47], much higher than it was the case in 1990 [48]. The political and economic instability in several Latin American countries (Brazil 1990, Mexico 1995, Ecuador 2000, Bolivia and Argentina 2002) [4] coincided with the largest increase in inequality (Gini Index) [5], all of which made it difficult to meet the objectives of the 2030 Sustainable Development Goals agenda [1]. Despite chronic political conflicts, liberalization policies have been consistently pursued. Since 1982, most of the Latin American countries applied structural adjustment policies, based on the guidelines of the “Washington Consensus”, including the control of public spending and fiscal deficits [49]. Frustration over inequality has resulted in massive mobilizations in the Latin American region in 2019. However, Ecuador joined the ranks of countries with apparent improvements in their average health indicators such as Chile and Colombia [47, 50].

### Inequalities in coverage over time - RMNCH interventions on a national level

The reduction of inequities in health services related RMNCH interventions between the periods 1994–2004 were not as evident as those between 2004 and 2012; which may be attributable to the prolonged period of political crisis faced between 1997 and 2006 as well as a financial crisis in 1999 [13, 15, 51]. In 2002, Ecuador allocated less than 5% of expenditure to public social spending (average in the region 15%) [52] and in 2005 it was one of the countries with the lowest public spending on health (only 5.9% of GDP) [17]. The decade between 1994 and 2004 was characterized by political instability [14], with a stagnation of the coverage of interventions occurring despite reported financial investments in the health sector [53]. However, programs like the Law on Free Maternity and Child Care from 1999 to 2004 were launched, and may have catalyzed improvements in primary healthcare services utilization and decreased neonatal mortality [54]. As these programs stabilized or were enhanced in the following period, gains from such interventions may have begun to emerge in the subsequent period.

Our results show that Ecuador remarkably reduced RMNCH intervention inequalities between 2004 and 2012, which is consistent with other studies and indicators that showed a reduction in inequalities in the use of health services after 2006 [22–24]. That could be related with the process of transformation of the health sector that started in 2007, reinforced by the Constitution of 2008 bolstering the protection of health as a human right [55]. Various actions were taken which reinforced the leadership of the Ministry of Public Health based on primary health care [17, 20, 22, 56]. Improvements in provisioning of health infrastructure, equipment and investment in medicine [17, 57]; increase in health human resources (the number of medical professionals per 10,000 inhabitants increased from 9.9 to 15.7 between 2008 to 2012) [58]. The benefits of these shifts in priority and investment appear to have had equity impacts: another study found that the percentage of live births attended by health personnel in rural areas (constant from 1990 to 2007), increasing 1.2 percentage points each year between 2008 to 2016 [59].

The reduction in inequalities has been associated with greater investment in education, more equitable social spending, and the implementation of social policies that support vulnerable population subgroups [5, 60, 61]. It is noted that the quality of governance measured by institutional capacity in the spending efficiency (bureaucracy, controls) for such reforms also plays a critical role [62].

The proportion of total expenditure on health as a proportion of GDP was 8.3% in 2014, increasing 2.8 percentage points between the period 2007 and 2014 [63]. Our analysis suggests that there may have been a positive and long-term influence of higher public spending on health coverage [64, 65] as has been observed in other Latin American countries [61]. This is in line with the established indirect relationship between public spending decline in the Gini index seen in the period 2004–2012 in several Latin American countries [60, 66, 67]. Overall, the increase in the total health expenditure as a percentage of GDP in Ecuador, together with other factors such as economic growth, poverty reduction [17, 68], improved access to education [69] and reduced income inequalities [70] may have contributed to this trend.

Early Initiation of breastfeeding in Ecuador nearly doubled between 2004 and 2012. This change may be attributable to the aforementioned spending, as well as a host of interventions specifically targeting breastfeeding, including the “Baby friendly Hospital initiative” [71], the Law on Promotion, Support and Protection of Breastfeeding [72] and compliance with the international code for the marketing of breast milk substitutes [73], reinforced in 2009, whose interventions can rapidly improve breastfeeding practices, when properly administered [74]. This intervention favored the poorest and rural populations, reflective of a global trend where more advantaged populations tend to have lower rates of breastfeeding coverage [7]. Regardless of these advances, the national coverage in 2012 remained low (around 50%) similar to countries like Peru or Bolivia [75].. Therefore, research and investment in this area should be expanded.

Despite the aforementioned overall improvements in RMNCH intervention, coverage in 2012 was still low compared to other countries such as Peru, Brazil and Colombia [76, 77], and inequality in favor of the wealthiest population subgroups persisted. Only the coverage use of modern contraceptive in 2012 was the intervention that achieved superior coverages compared to Peru, Mexico, Bolivia, Guatemala, Colombia and Argentina [28, 78]; indicating a success in intervention strategies which should be further analyzed in future studies.

### Inequalities in coverage over time – WAS interventions on a national level

Access to improved water sources and sanitation facilities also influence women’s and children’s health and their ability to receive essential interventions [79]. Ecuador did not show improvements over time, in these coverage indicators, despite the fact that the 2008 Constitution promulgated access to public services, education, employment and healthy environments [55]. While Ecuador joins other countries in the region that have shown significant improvement in sanitary facility coverage over time [80], it has not been equitable. For *improved sanitary facilities,* the greatest increase was from 1994 to 1999, likely attributable to the FASBASE project [53] which started in 1992 to support projects of basic services and sanitation nationwide. Unfortunately, 1999 onwards, this intervention stagnated, with coverage reducing by 2012 in many provinces, except for those whose local governments prioritized this intervention. Interestingly, while coverage for the WAS indicators was 80% nationwide, it was only around 50% for the poorest quintiles, suggesting marginal exclusion. Other studies are consistent with these findings, since they show that Ecuador in 2015 was among the countries with relatively lower degrees of concentrated poverty, but great inequalities in access to drinking water and sanitation regardless [81].

According to the Constitution of the Republic of Ecuador, it is the responsibility of municipal governments to provide the public with drinking water, sewerage, sewage treatment, solid waste management and environmental sanitation activities established by law [55].. Health concerns are a shared responsibility between the ministries of health and social areas, as well as the municipal governments, given the direct impact on the health of the population, especially the most vulnerable. This analysis suggests that greater attention and intersectoral action is clearly needed to reduce geographic inequalities in drinking water and sanitation access [79] which has the potential to bring about lasting and positive change for women, children, families and communities [32]..

Overall, the achieved national average is not a sufficient indicator of the country’s progress in terms of health. Intervention coverage distribution across population subgroups must be assessed to identify vulnerable population and reduce inequality [8, 11]. Additionally, lessons could be drawn from provinces with successful intervention outcomes over time, to see whether their strategies are applicable to similar populations.

### Inequalities in coverage interventions over time on a sub-national level

Although previous studies conducted with data from 2004 and 2009 showed no difference in access to health services by provinces [18, 23], the present study extends the analysis to three different points in time, observing that the differences between provinces in 1999 and 2004 were greater compared to the differences observed in 2012, that is, the coverage achieved at the national level in this year, were also distributed more equitably at the subnational level. This is consistent with previous studies that showed an improvement in coverage over time that has favored vulnerable areas [39].

Different inequality patterns where seen by geographic regions. For instance, Pichincha the province in the mountain region that includes Quito, the capital of Ecuador shows consistently high coverage compared to other mountain provinces with high degrees of poverty, such as Bolivar, Chimborazo and Cotopaxi. These provinces share the lowest coverage of interventions due to a number of characteristics like large percentage of rural population, high rates of illiteracy, high total fertility rates, and they also present the lowest rate of doctors per 10,000 inhabitants [58, 82]. In the Amazon region, the greatest improvement in the coverage were observed in the provinces that had low intervention coverage in 1999 [82]. Most of the provinces of the Amazon region have low Gross Domestic Product (GDP) per capita [83], with large rural, indigenous, and relatively less educated population groups [82], Despite this improvements, 2012 coverage levels within this region remained comparatively low. In fact between 1995 and 2006, a pronounced increase in poverty and social inequality in rural households was observed in Paramos of the central highlands and the dispersed colonized areas of the Amazon [45].

This study did not identify significant regional patterns with exception of the breastfeeding intervention which is related with factors such as rurality and indigenous belonging [84]. More consistent information – by ethnic status and other proxies for social determinants together with a higher level of geographic disaggregation - is needed given that these factors prevail in smaller subpopulations within provinces. This would allow for the identification of regional patterns across provincial borderlines as province boundaries also mask internal inequalities.

### Strengths and limitations of the study

Adding to the literature on health inequalities in Ecuador [22, 23, 85] the present study compares coverages of RMNCH and WAS indicators using a broad time-frame of analysis. Critically, data analysis was disaggregated by subnational level using geographic information systems for mapping, which is especially important for Ecuador, which has four socially and structurally distinct regions. The present study can provide useful lessons to other countries on the effectiveness of comprehensive analyzes with geographical and temporal disaggregation.

However, the reporting periods of data in this survey were not equidistant – i.e. between 1994, 1999 and 2004, there was a gap of 5 years each, but between 2004 to 2012, the gap was 8 years. Such analyses could reveal clearer insights if the periodicity of surveys were fixed. Nevertheless, available information provides a reasonable basis for the present analysis. This study analyzes data up to the year 2012, but it is possible that other changes have occurred in a more current period, which should be evaluated when more recent surveys are available.

To achieve comparability throughout the four surveys, all indicators used in the present analyses have been standardized at the International Center for Equity in Health (ICEH; www.equidade.org).

It was not possible to include more coverage indicators, as information to calculate them was limited. In the *full immunization coverage* indicator*,* a change was recorded in the vaccination schedule among those reported by the 1999 survey and the 2004 survey, which limits comparability [27]. Moreover, the indicator *improved drinking water source* showed a change in the elements forming the indicator for 2012, but this did not affect the result when it was compared with official reports [28]. However, in the breakdown by province, due to the weakness in this indicator, we considered it better to disaggregate only the indicator *Improved Sanitary Facilities* which showed a similar behavior between provinces and the same conclusion was reached. Finally, with regard to the indicator on *early initiation of breastfeeding,* the survey of 2012 related questions were referring only to children younger than 24 months whereas in the 2004 survey they were referring to children under 5 years [28]. Therefore, these three indicators should be interpreted with caution.

Inequality analysis by geography is not easy to measure in national surveys, especially due to sampling problems that do not allow for a very fine geographic stratification [35]. For the first time we have analyzed intervention coverage of RMNCH geographically disaggregated by provinces in the indicated time period. It must be noted, however, that most official survey reports suggest not considering data of the Amazonian region disaggregated by provinces, due to the lack of statistically representative samples [25, 26], with exception of the last survey 2012 [28]. It was also difficult to obtain high-quality information in this region, which must also be interpreted with caution. We need solid evidence on subnational health inequalities and other social and cultural determinants to improve the analysis.

It was beyond the scope of this analysis to examine the coverage of RMNCH interventions in indigenous population using ethnicity as a dimension of inequality which is recommended by international organizations and other studies [86, 87]. Analysis related to this sub-group (which overlaps with those in the poorest quintiles, living in rural areas), is currently underway.

## Conclusions

Ecuador is a country that has endured economic and political crises, and yet has achieved progress in both RMNCH coverage of interventions, as well as reducing inequalities between 1994 and 2012. In 2012 inequalities in RMNCH health service-related interventions show a more marked reduction, compared to the previous decade (1994–2004). These reductions in inequality coincided with regained political stability, the promotion of redistributive policies, and greater social spending, different from the neo-liberal reforms that had been applied for more than 20 years before. These changes together with other factors such as improvements in economic conditions and reduction of poverty may have had an important positive impact on health inequality. In contrast, inequalities in coverage of basic sanitation and drinking water remained high. Notwithstanding improvement at the subnational level, geographic inequality persists in Ecuador and warrants further study. Policy and research attention should also turn to understanding and acting on the nature and causes of these inequalities so that Ecuador may join other countries on the UHC path of “leaving no one behind” [88].

## Supplementary Information

**Additional file 1.**

## Data Availability

All data used in this study are available in the repository of the World Bank database (surveys of the years 1994, 1999 and 2004) link: http://microdata.worldbank.org/index.php/catalog/?country[]=ecuador; and survey of 2012 in the National Institute of Statistics and Censuses of Ecuador (INEC), https://www.ecuadorencifras.gob.ec/encuesta-nacional-de-salud-salud-reproductiva-y-nutricion-ensanut-2012/, which have no restrictions to its use by non-academics.

## Abbreviations

RMNCH: Interventions reproductive, maternal neonatal and children
WAS: Interventions of drinking water sources and improve sanitary facilities
ENSANUT: Encuesta nacional de salud y nutrición
RHS: Reproductive health survey
SII: Slope index of inequality
CIX: Concentration index
LMGAI: Ley de maternidad gratuita y atención a la infancia
INEC: Instituto nacional de estadísticas y censos
WHO: World health Organization
PAHO: Panamerican health Organization
