# Supplementary material for: National and subnational coverage and inequalities in reproductive, maternal, newborn, child, and sanitary health interventions in Ecuador: a comparative study between 1994 and 2012
Source: Int J Equity Health. 2021 Jan 28;20:48. doi: 10.1186/s12939-020-01359-1 (PMC7842066; doi:10.1186/s12939-020-01359-1)
Supplement: Supplementary file 1 — Additional file 1. [file 12939_2020_1359_MOESM1_ESM.docx]

**Supplementary annex 1**

| **Table 1. List of Indicators and calculate made with standardized definitions** |
| --- |

| **Categories** | **Indicator name** | ***Indicator denominator*** | ***Indicator numerator*** | ***Interpretation*** |
| --- | --- | --- | --- | --- |
| Sexual and reproductive health  *(RMNCH)* | Current use of any contraceptive method (CPMO) | Women aged 15-49 years currently married or in union | Who are using (or whose partner is using) any method | *Percentage of women between the ages of 15 and 49 who are currently married or in common union, using a modern contraceptive method* |
| Antenatal care  *(RMNCH)* | Antenatal care (four or more visits) | Women 15-49, live birth in the last 3/5 years, last-born child | 4+ visit(s) with skilled provider | *Percentage of women aged 15 to 49 years whose last child was born 3-5 years ago, who made 4 or more visits during pregnancy with trained professionals* |
| Delivery assistance  *(RMNCH)* | Institutional delivery | All live births in the last 2 years | Delivered in a health facility | *Percentage of live births in the last 2 years that were attended in a health institution* |
| Child health  *(RMNCH)* | Full immunization coverage | Livechildren, 12-23/18-29/15-26 months | 3 doses of DPT & 3 doses of Polio & received Measles & received BCG | *Percentage of children up to 26 months of age who received detailed vaccine doses* |
| Breastfeeding  *(RMNCH)* | Exclusively breastfeeding | Last born, living with respondents, specific age groups | Breastfed exclusively (only breastmilk) | *Percentage of live births who, up to 6 months, received only exclusive breastfeeding* |
| Water and Sanitary interventions (WAS) | Improved drinking water access | All household members | With access to an improved source of drinking water in the household | *Percentage of households that have access to household water sources* |
| Water and Sanitary interventions (WAS) | Improved sanitation | All household members | With improved sanitation (non-shared) facility | *Percentage of households that have access to sanitary improvements* |

**Supplementary annex 2:**

An analysis of complex inequality measures was carried out with data from the 2012 survey following the recommendations of the literature (1). We calculated two absolute summary measures of inequality, the difference of Q1 and Q5 (Q5 – Q1) and the Slope Index of Inequality (SII) and two relative measures, the ratio of Q1 vs. Q5 (Q5:Q1) and the Concentration Index (CIX) (2,3). The SII takes all the subgroups into consideration, not only the most- advantaged and the most- disadvantaged (using regression model); the value zero is interpreted as no inequality while positive values indicate higher coverage in the advantaged subgroups and negative values indicate higher coverage in the disadvantaged subgroups. The relative measure based on a ratio of Q1 vs. Q5 means that if health services coverage were 100% and 50% in two subgroups, this would equal 2 times higher coverage in the richest compared to the poorest (3) subgroups. The CIX is related to the Gini coefficient, where 0 implies an absence of inequality, values ​​ between -1 and 1 imply favorable indicators, negative values ​​imply greater intervention coverage among the poor and positive values ​​imply greater coverage among the rich (1). All measures were calculated with the 95% confidence interval and were multiplied by 100 for easy interpretation.

In 2012, median coverage of interventions was 79%, and for most of the interventions the interquartile range was 69% to 87%. Between RMNCH interventions, *the ratio Q5:Q1 for Antenatal care 4+ visits and institutional delivery was 1.3, that means* rich people had 1.3 times greater coverage than the poorest (CIX 5.1; 95%; CI 4.6-5.7% and CIX 6.7; 95%; CI 6.3-7.2%, respectively).  *Early initiation of breastfeeding* showed significant inequality favoring the poorest quintiles (CIX -4.5; 95%; CI -6.0- -3.1%). (see Table 1).

The greatest inequality was observed in WAS interventions. *Improved sanitary facilities* and *drinking water sources* had average coverage levels of 78.8% and 85.6% respectively, but when disaggregating the information by wealth quintiles, only around 50% of the poorest quintile had coverage, indicating a wide gap between rich and poor.

In 2012, the ratio Q5:Q1 of *improved sanitary facilities* was 2.4, that means individual whose incomes were in the highest quintile had 99.1% of coverage while the poorest quintile had 44% or in other words, rich people had 2.4 times greater coverage in improved sanitary facilities than the poorest (CIX 14.9; 95%; CI 13.8-16.0%). Similar results were observed for the *improved drinking water* indicator where coverage was 2.0 times greater in the wealthiest quintile. Complex measures (SII and CIX), which take into account the values of intermediate quintiles, showed larger, significant magnitudes of inequality than simple measures (which are calculated using just the extreme quintiles for all indicators) in all indicators except *for full immunization coverage*. (see Table 1)

**Supplementary annex 2:**

| **Table 1. Coverage and magnitude of inequalities by intervention in Ecuador 2012** | | | | | | | | | | | | | | |  |  |  |  |  |  |  |  |  | |  | |  |  |  |  |  | |  | |  | |  |  |  |  |  | |  | |
| --- | --- | --- | --- | --- | --- | --- | --- | --- | --- | --- | --- | --- | --- | --- | --- | --- | --- | --- | --- | --- | --- | --- | --- | --- | --- | --- | --- | --- | --- | --- | --- | --- | --- | --- | --- | --- | --- | --- | --- | --- | --- | --- | --- | --- |
| **Intervention** | **Overall  Coverage % (CI 95%)** | | | | |  | **Quintile 1  Coverage % (CI 95%)** | | | | |  | **Quintile 5  Coverage % (CI 95%)** | | | | |  | **Difference Q5-Q1  * (CI 95%)** | | | | |  | | **Slope Index of Inequality * (CI 95%)** | | | | | |  | | **Ratio (Q5:Q1)** | | **Concentration Index  x100 (CI95%)** | | | | | |  | |  |
| **Use of modern contraceptive** | **70.9** | ( | 69.2 | - | 72.5 | ) | **66.6** | ( | 62.3 | - | 70.6 | ) | **71.9** | ( | 68.9 | - | 74.7 | ) | **5.3** | ( | 2.6 | - | 8.0 | | ) | | **7.3** | ( | 3.0 | - | 11.6 | | ) | | **1.1** | | **3.6** | ( | 3.0 | - | 4.3 | | ) | |
| **Antenatal care 4+ visits** | **86.8** | ( | 84.9 | - | 88.5 | ) | **75.0** | ( | 71.0 | - | 78.6 | ) | **96.0** | ( | 92.8 | - | 97.8 | ) | **21.0** | ( | 18.8 | - | 23.2 | | ) | | **24.7** | ( | 19.1 | - | 30.3 | | ) | | **1.3** | | **5.1** | ( | 4.6 | - | 5.7 | | ) | |
| **Institutional delivery** | **90.2** | ( | 88.0 | - | 92.1 | ) | **76.4** | ( | 70.6 | - | 81.3 | ) | **98.5** | ( | 96.9 | - | 99.3 | ) | **22.1** | ( | 20.5 | - | 23.8 | | ) | | **31.1** | ( | 23.0 | - | 39.3 | | ) | | **1.3** | | **6.7** | ( | 6.3 | - | 7.2 | | ) | |
| **Early initiation of breastfeeding** | **52.6** | ( | 49.7 | - | 55.5 | ) | **61.7** | ( | 56.4 | - | 66.7 | ) | **44.0** | ( | 39.5 | - | 48.7 | ) | **-17.7** | ( | 23.1 | - | -12.2 | | ) | | **-22.9** | ( | 28.5 | - | 17.4 | | ) | | **0.7** | | **-4.5** | ( | -6.0 | - | -3.1 | | ) | |
| **Full immunization coverage** | **66.6** | ( | 62.9 | - | 70.1 | ) | **63.1** | ( | 56.0 | - | 69.6 | ) | **74.0** | ( | 64.4 | - | 81.7 | ) | **10.9** | ( | 4.0 | - | 17.8 | | ) | | **8.0** | ( | -3.0 | - | 19.1 | | ) | | **1.2** | | **4.2** | ( | 2.4 | - | 6.1 | | ) | |
| **Improved sanitary facility** | **78.8** | ( | 76.6 | - | 80.9 | ) | **41.0** | ( | 37.5 | - | 44.6 | ) | **99.1** | ( | 98.4 | - | 99.4 | ) | **58.1** | ( | 56.6 | - | 59.5 | | ) | | **67.8** | ( | 64.8 | - | 70.9 | | ) | | **2.4** | | **15.4** | ( | 15.0 | - | 15.9 | | ) | |
| **Improved drinking water source** | **85.6** | ( | 82.8 | - | 88.1 | ) | **49.8** | ( | 45.3 | - | 54.2 | ) | **99.7** | ( | 99.1 | - | 99.9 | ) | **50.0** | ( | 48.5 | - | 51.4 | | ) | | **62.9** | ( | 57.9 | - | 67.8 | | ) | | **2.0** | | **10.9** | ( | 10.5 | - | 11.3 | | ) | |

*Sources:* Ecuador ENSANUT 2012. * percentual point

**Supplementary annex 3:**

**Figure 1.** Trend of four intervention coverage by province (A-E). Ecuador 1994 to 2012.


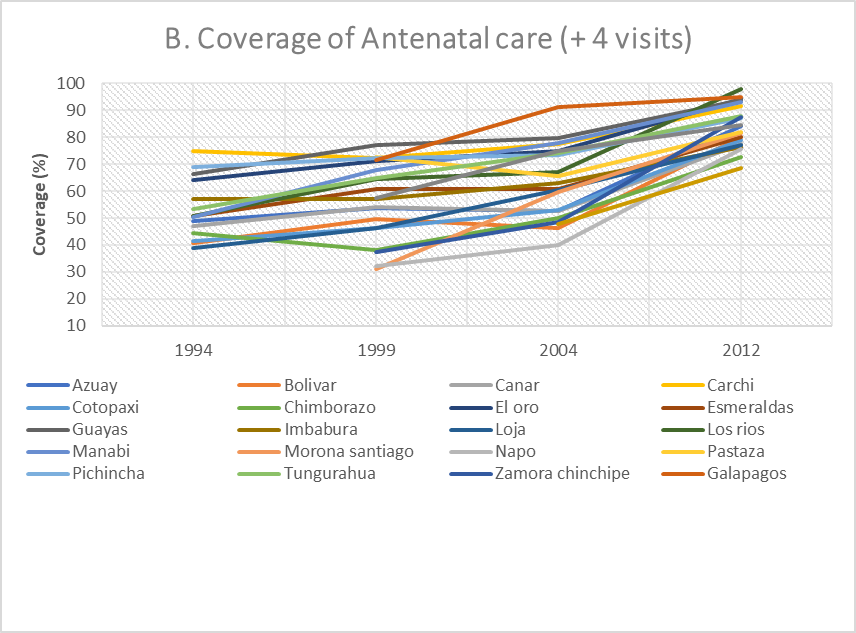

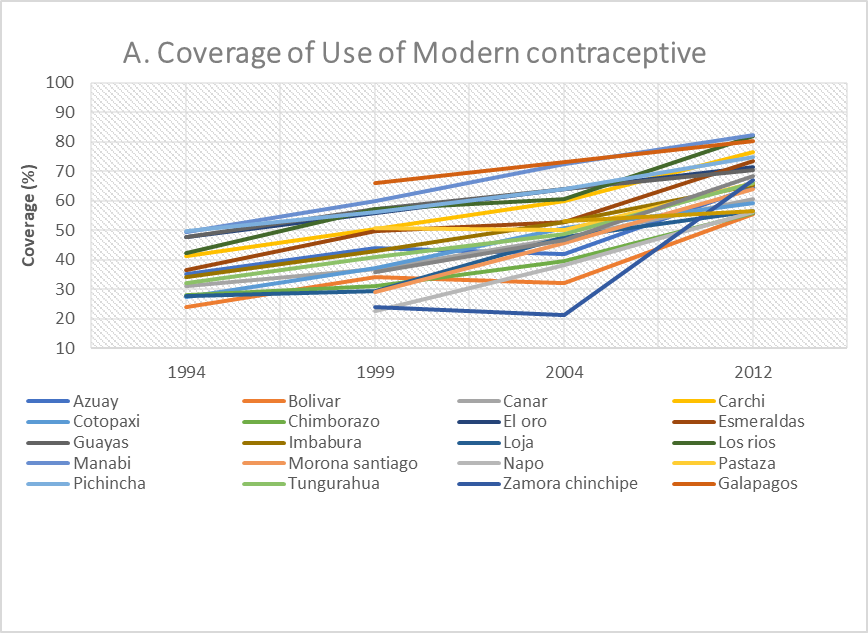


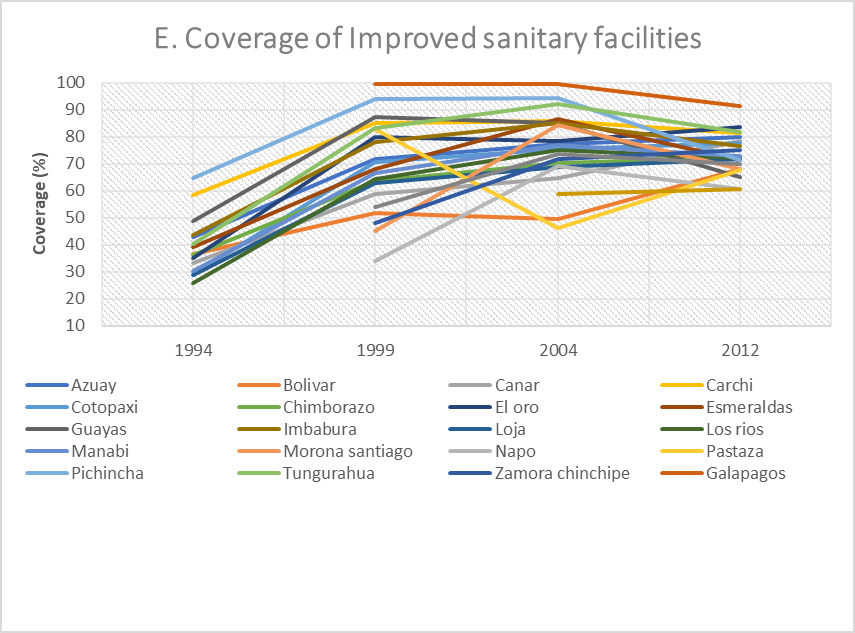

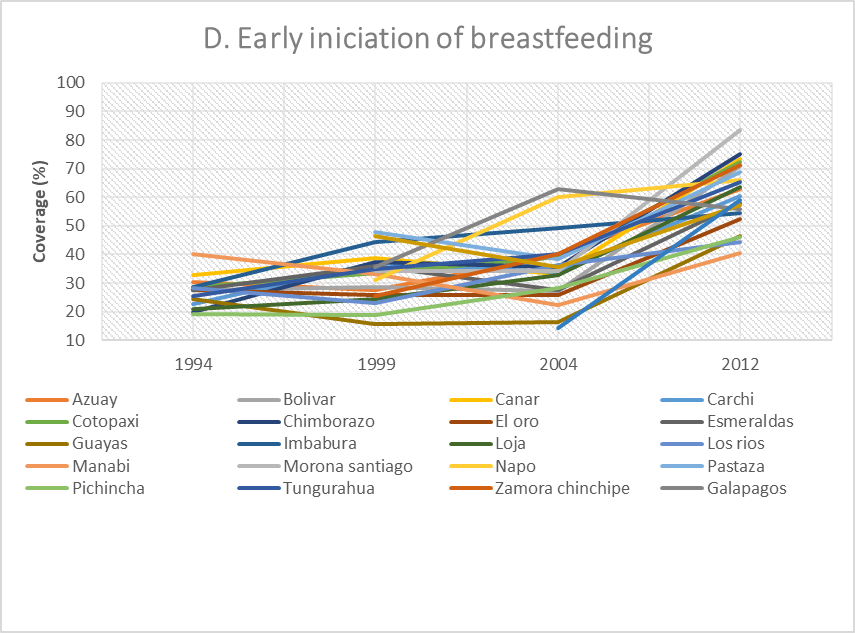

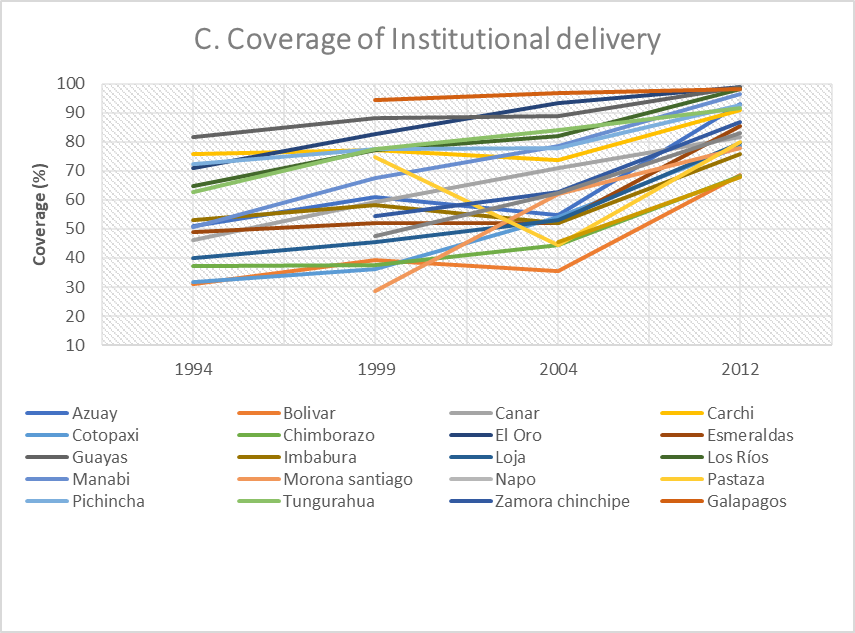


Sources: National health survey 1994, 1999, 2004, 2012.

**Supplementary annex 4:**

**Figure 2.** Average annual absolute change in percentage points by province and intervention (A-E). Ecuador 1994- 2012.


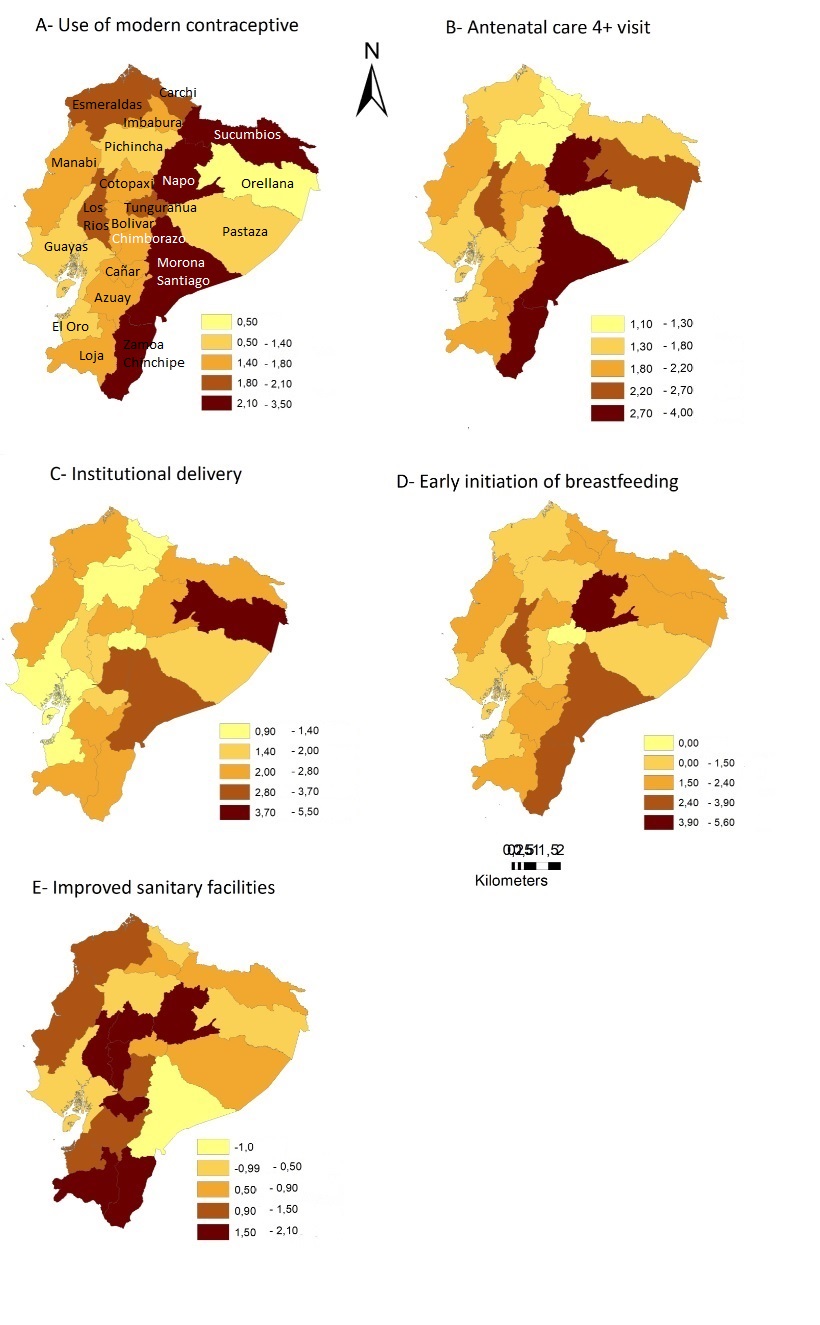


*Sources:* Ecuador RHS 1994, RHS 1999, RHS 2004, ENSANUT 2012

**Bibliography:**

1. Barros AJD, Victora CG. Measuring Coverage in MNCH: Determining and Interpreting Inequalities in Coverage of Maternal, Newborn, and Child Health Interventions. PLOS Med. 7 de mayo de 2013;10(5):e1001390.

2. O’Donnell O, van Doorslaer E, Wagstaff A, Lindelow M. Analyzing health equity using household survey data : a guide to techniques and their implementation [Internet]. The World Bank; 2007 nov [citado 14 de mayo de 2019] p. 1-234. Report No.: 42480. Disponible en: http://documents.worldbank.org/curated/en/633931468139502235/Analyzing-health-equity-using-household-survey-data-a-guide-to-techniques-and-their-implementation

3. PAHO/WHO. Manual para el Monitoreo de las Desigualdades en Salud, con especial énfasis en países de ingresos medianos y bajos [Internet]. Biblioteca sede de la OPS. Washington D.C.; 2016 [citado 14 de noviembre de 2016]. Disponible en: http://www.paho.org/hq/index.php?option=com_content&view=article&id=12571%3Amanual-monitoreo-desigualdadessalud-paises-ingresos-medianos-bajos&catid=8896%3Apublications&Itemid=42134&lang=es
